# Delayed Diagnosis of Severe Hypoglycemia in a Septic Patient With Chronic Renal Failure

**DOI:** 10.7759/cureus.28615

**Published:** 2022-08-31

**Authors:** Daan Ten Berge, Fokko Manning, Vera Silderhuis, Saskia Deijns, Marie-Jose Pouwels, Hans Krabbe, Albertus Beishuizen

**Affiliations:** 1 Department of Clinical Chemistry and Laboratory Medicine, Medisch Spectrum Twente, Enschede, NLD; 2 Intensive Care Center, Medisch Spectrum Twente, Enschede, NLD; 3 Department of Internal Medicine, Medisch Spectrum Twente, Enschede, NLD; 4 Department of Clinical Chemistry and Laboratory Medicine, Medlon BV, Enschede, NLD

**Keywords:** vitamin c, blood glucose meters, point-of-care, interference, hypoglycemia, glucose

## Abstract

High-dose vitamin C therapy has gained increased interest as an adjunctive treatment of septic shock, although convincing evidence is still lacking. High blood levels of vitamin C may interfere with several point-of-care blood glucose meters. We describe the case of a 67-year-old septic patient known with chronic renal failure who developed truly severe hypoglycemia, which was masked by spuriously high glucose values measured on a capillary blood glucose meter. This initially led to the treatment of spurious hyperglycemia with high-dose insulin and a delayed correct diagnosis and treatment, rendering substantial risk for the patient. Awareness of this dangerous interference is warranted.

## Introduction

High-dose vitamin C therapy can be used as an adjunctive treatment of septic shock [1]. High blood levels of vitamin C may interfere with several point-of-care blood glucose meters (BGMs) [2,3]. BGMs are well embedded in the routine hospital care for rapid assessment of glucose homeostasis. Despite many advantages of the use of these point-of-care testing (POCT), unawareness of the limitations and interferences may introduce incorrect diagnosis and/or treatment. We describe a 67-year-old septic patient who developed a coma due to severe hypoglycemia, which was initially masked by factiously high glucose values measured on the Accu-Chek Inform II (ACI II, Roche Diagnostics, The Netherlands) BGM. This led to insulin treatment of the artificial hyperglycemia deepening the hypoglycemia and delaying the correct diagnosis and treatment of the severe hypoglycemia, rendering substantial risk for the patient.

## Case presentation

A 67-year-old female with a medical history of chronic renal failure on hemodialysis and peripheral vascular disease was postoperatively admitted to our intensive care unit (ICU) after percutaneous transluminal angioplasty of the left femoral superior artery, popliteal artery and tibialis anterior artery combined with a hallux amputation because of an infected necrotizing foot ulcer. Initially, she was in need of norepinephrine (vasopressor) due to septic shock. On the second postoperative day, circulatory failure deepened and she was treated with high-dose norepinephrine, piperacillin-tazobactam in combination with a high dose of vitamin C (1,500 mg, four times daily), hydrocortisone (50 mg, four times daily) and thiamine (200 mg, two times daily). The source of sepsis was infected necrosis of the left foot which was treated surgically. The patient was treated with intravenous continuous insulin aspart (Novorapid) infusion to maintain glucose levels between 6 and 8 mmol/L (108-144 mg/dL). Intermittent hemodialysis (IHD) was performed on day 3 without any problem. Gradually, vasopressor support was tapered, and on day 5, she was discharged to the surgical ward. At the time of discharge from the ICU, she was still treated with high-dose vitamin C, hydrocortisone, thiamine and intravenous continuous Novorapid infusion (8 U/h).

In the surgical ward, she suddenly developed acute symptoms of aphasia and a decreased level of consciousness with a temperature of 36.2°C, a blood pressure of 110/50 mmHg, a pulse rate of 81/min and a pulse oxygen saturation of 95%. The capillary glucose concentration was 5.9 mmol/L (106 mg/dL), measured using the Accu-Chek Inform II (ACI II, Roche Diagnostics, The Netherlands), which is based on the electrochemical detection of glucose using a specific glucose dehydrogenase enzyme. Brain computed tomography (CT) was negative for intracranial hemorrhage or infarction. Due to progressive arterial hypotension and coma, the patient was readmitted to the ICU on the same day with a clinical diagnosis of brain ischemia. Plasma glucose measurement was performed on a routine chemistry analyzer (Cobas 8000, Roche Diagnostics, The Netherlands). The glucose concentration was found to be 0.9 mmol/L (16.2 mg/dL). Novorapid was discontinued, and glucose infusion was started. We immediately retested glucose concentrations both on ACI II and the routine chemistry analyzer at the central lab which showed a value of 6.6 (119 md/dL) and 2.1 mmol/L (38 mg/dL), respectively. Moreover, a whole blood glucose measurement was performed at the same time with an ABL90 blood gas analyzer (Radiometer, Denmark), which displayed a similar glucose concentration as the routine chemistry analyzer. After prompt correction of the severe hypoglycemia, consciousness rapidly improved.

Due to vitamin C interference, a marked discrepancy was observed between the capillary blood glucose measured on ACI II and the glucose concentration of the venous sample measured on the routine chemistry analyzer, which was confirmed by glucose spiking experiments described elsewhere [2]. The use of the BGM in this specific patient was discontinued, and all glucose measurements from that moment onward were performed using the ABL90 equipment.

**Figure 1:**
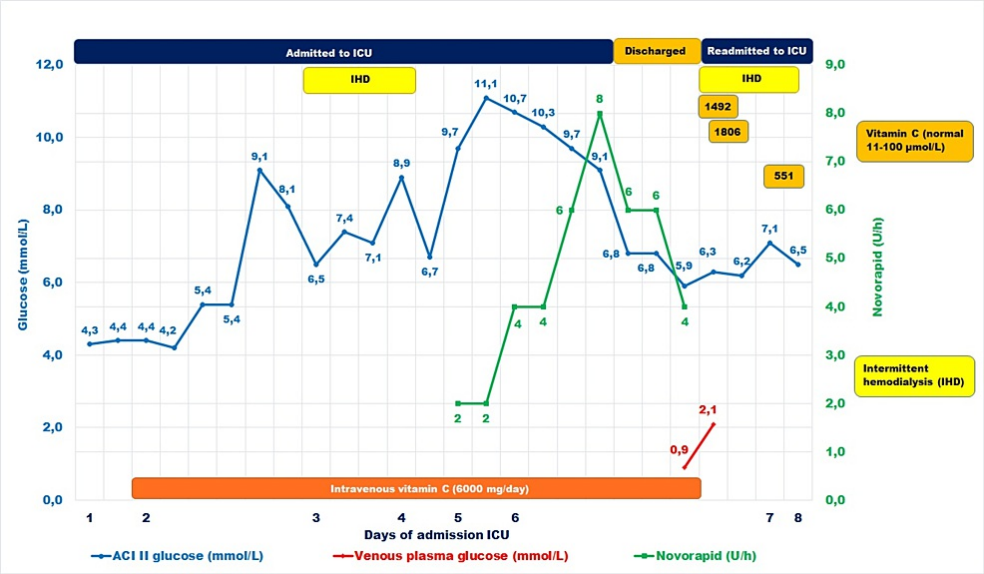
Biomarker concentrations and relevant clinical data during the admission of the patient IHD: intermittent hemodialysis, ACI II: Accu-Chek Inform II.

Residual plasma from the time of the low glucose measurement was used to measure vitamin C, which demonstrated an extremely high level of 1,492 µmol/L (reference range: 11-100 µmol/L), probably due to renal failure. Moreover, two hours after the detection of hypoglycemia, the vitamin C level increased to 1,806 µmol/L. Forty hours after discontinuing vitamin C treatment and reinitiating IHD, the concentration decreased to 551 µmol/L. She continued to improve clinically, maintained euglycemia and was discharged seven days after the initial ICU admission. In Figure 1, we summarize the time course of glucose concentrations, insulin dosage and clinical interventions.

## Discussion

Septic patients develop multiple organ dysfunction induced by ischemia/reperfusion injury or distributive shock. Overwhelming amounts of reactive oxygen species (ROS) and reactive nitrogen species are generated under these conditions, and ROS-mediated microcirculatory impairment and endothelial dysfunction may occur [4]. Preclinical and small clinical studies have shown that high-dose vitamin C may aid in modulating ROS-induced microcirculatory flow disturbances and vascular responsiveness mainly by protecting against oxidative stress [4]. The biological plausibility and several small clinical observational trials of vitamin C therapy (most often in combination with thiamine and hydrocortisone) prompted clinical application in several ICUs worldwide [5,6]. However, these remarkable results have not consistently been validated in more recent randomized controlled trials (RCTs) [7,8]. Moreover, vitamin C therapy has been associated with adverse effects [9], and therefore, controversy continues to exist [6].

Based on the protocol by Marik et al. [5], which has been adopted in our ICU, most patients with sepsis receive a high dose of vitamin C (1,500 mg, four times daily), hydrocortisone (50 mg, four times daily) and thiamine (200 mg, two times daily). Despite the manufacturer's warnings in the package insert of the product, physicians were not fully aware that these high doses of vitamin C could hamper glucose interpretations when measured with our capillary BGM.

Our case report demonstrates a dangerous and potentially life-threatening interference of vitamin C on our capillary BGM in a patient receiving high-dose vitamin C therapy and in particular with renal failure. Several studies have shown that vitamin C can cause falsely elevated point-of-care glucose measurements due to not only interference electrochemical method but also calorimetric analytic method, potentially displaying hypoglycemia in patients with renal failure treated with high doses of vitamin C [10,11]. Spiking experiments using the blood of three healthy volunteers were spiked with vitamin C with increasing concentration, providing proof that these high concentrations of vitamin C indeed resulted in falsely elevated glucose concentrations on our BGM [2]. BGM measurements leading to factitiously elevated glucose levels and consequently to the administration of excessive and unnecessary insulin may induce or aggravate hypoglycemia and delayed diagnostics.

## Conclusions

High levels of vitamin C in blood interfere with several BGMs (both calorimetric and electrochemical methods); therefore, extreme caution is warranted. The vitamin C interference on the BGMs will lead to overestimation of blood glucose and potentially mask hypoglycemia. Renal failure, as in our patient, will decrease the vitamin C clearance and increase the risk of misdiagnosing hypoglycemia.

Therefore, awareness of this potential interference of vitamin C and glucose measurements on BGMs is warranted and close collaboration of intensivists and clinical chemists may prevent complications such as those presented in our case report. Automated signaling by clinical decision rules could have prevented this adverse treatment. Potentially dangerous hypoglycemia can be prevented by using conventional laboratory glucose measurement or BGMs that do not display this interference.
